# A mAb against surface-expressed FSHR engineered to engage adaptive immunity for ovarian cancer immunotherapy

**DOI:** 10.1172/jci.insight.162553

**Published:** 2022-11-22

**Authors:** Devivasha Bordoloi, Pratik S. Bhojnagarwala, Alfredo Perales-Puchalt, Abhijeet J. Kulkarni, Xizhou Zhu, Kevin Liaw, Ryan P. O’Connell, Daniel H. Park, Daniel W. Kulp, Rugang Zhang, David B. Weiner

**Affiliations:** 1 Vaccine & Immunotherapy Center and; 2 Immunology, Microenvironment and Metastasis Program, The Wistar Institute, Philadelphia, Pennsylvania, USA.

**Keywords:** Oncology, Therapeutics, Adaptive immunity, Cancer, Immunotherapy

## Abstract

Despite advances in ovarian cancer (OC) therapy, recurrent OC remains a poor-prognosis disease. Because of the close interaction between OC cells and the tumor microenvironment (TME), it is important to develop strategies that target tumor cells and engage components of the TME. A major obstacle in the development of OC therapies is the identification of targets with expression limited to tumor surface to avoid off-target interactions. The follicle-stimulating hormone receptor (FSHR) has selective expression on ovarian granulosa cells and is expressed on 50%–70% of serous OCs. We generated mAbs targeting the external domain of FSHR using in vivo–expressed FSHR vector. By high-throughput flow analysis, we identified multiple clones and downselected D2AP11, a potent FSHR surface–targeted mAb. D2AP11 identifies important OC cell lines derived from tumors with different mutations, including BRCA1/2, and lines resistant to a wide range of therapies. We used D2AP11 to develop a bispecific T cell engager. In vitro addition of PBMCs and T cells to D2AP11-TCE induced specific and potent killing of different genetic and immune escape OC lines, with EC_50_s in the ng/ml range, and attenuated tumor burden in OC-challenged mouse models. These studies demonstrate the potential utility of biologics targeting FSHR for OC and perhaps other FSHR-positive cancers.

## Introduction

Ovarian cancer (OC) represents the deadliest gynecologic malignancy. It stands as the fifth major driver of deaths from cancer among women, accounting for the highest number of deaths for cancer of the female reproductive system. The American Cancer Society estimated that there were 21,410 women with a new OC diagnosis and 13,770 deaths due to OC in 2021 (1, 2). OC is a highly heterogeneous cancer where 90% of tumors are of epithelial origin. The most prevalent subtype of epithelial OC is high-grade serous cancer, constituting around 70% to 80% of cases, whereas low-grade serous (<5%), endometrioid (10%), clear cell (10%), and mucinous (3%) represent less predominant subtypes (3).

Surgery and chemotherapy are the primary treatments for OC (4). These approaches are partially successful, with many patients developing chemoresistance within a few years after their initial treatment, who are next faced with disease recurrence (4). High mortality from OC is also linked to low rates of early detection, often due to the lack of subjective symptoms as well as marginally invasive techniques for primary detection. Therefore OC remains a critical need area for novel therapeutic approaches (5). There is a close interaction between the ovarian tumor cells and the tumor microenvironment; thus, development of treatment approaches that not only target the tumor cells but also can maintain their antitumor function in this microenvironment is of particular importance (6). A growing area of study is immune-based therapies for OC, including immune checkpoint inhibitors and T cells engineered from chimeric antigen receptors (CARs) and T cell receptors (4). Notably, a major obstacle in the development of CAR therapies is to find targets with specific expression confined to the surface of tumor cells and not on healthy tissues (7). The follicle-stimulating hormone receptor (FSHR) is one important target reported to have selective expression on ovarian granulosa cells versus low levels of expression on the normal ovarian endothelium. FSHR is expressed in 50%–70% of serous ovarian carcinoma cases, providing an important potential target for immune therapies (7).

mAbs are an important tool in the diagnosis, classification, treatment, and monitoring of specific cancers. Some examples include anti-HER2 antibodies in various forms for the classification and treatment of breast cancer (8, 9), anti-CD20 antibodies for therapy of lymphoma (10), anti-CA125 for the follow-up of OC (11), and anti-PSA for detection of prostate cancer (12). A recent area of importance in the field of antibody therapeutics has been studies of bispecific T cell engagers (TCEs) (13–18). These represent a novel class of immunotherapy with the ability to bind both T cells and tumor cells simultaneously in order to facilitate the cytolytic function of T cells against specific tumor cells (19, 20). Such a tool for OC would be important for additional study.

In this report, we describe the development of monoclonal reagents as tools to target FSHR as a tumor-associated antigen of importance in OC. We characterized these and used them to develop a bispecific TCE (FSHRxCD3). It was then evaluated for its ability to specifically redirect T cells to OC in vitro and to impact OC in therapeutic mouse models in vivo. We report that this can be a specific and effective tool for killing of OC. These data demonstrate the therapeutic potential of directly targeting OC through the engagement of the adaptive immune system.

## Results

### Generation and flow cytometry screening of anti–human FSHR antibodies.

**Figure 1:**
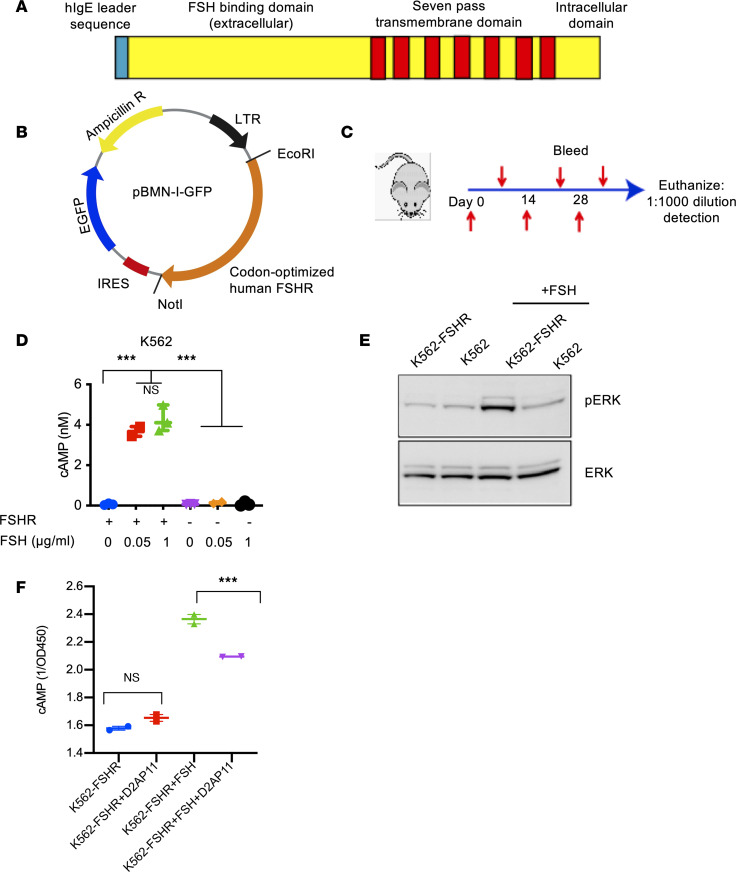
Generation of anti-human FSHR antibodies. (**A**) Depiction of FSHR structure. (**B**) Cloning strategy into pBMN-I-GFP expression vector. (**C**) Mouse immunization scheme. (**D**) cAMP response to different doses of FSH of K562 and K562-FSHR. (**E**) Western blot of phospho-p44/42 (Erk1/2) and p44/42 (Erk1/2) 20 minutes after stimulation of K562 and K562-FSHR cells using FSH. (**F**) Partial block of cAMP production in K562-FSHR cells by D2AP11 anti-FSHR antibody upon FSH stimulation of FSHR. Error bars represent mean ± SEM; all the experiments were done in duplicate or triplicate. ANOVA. ***P < 0.001.

FSHR is a tumor-associated antigen present in OC (7), prostate cancer (21), and the neovessels of 80% of cancers (22). It is a G protein coupled with 7 transmembrane domains (23). This complex structure presents challenges for classical antigen protein approaches. We generated a codon-optimized sequence of the human FSHR (Figure 1A) for direct in vivo immunization allowing for the generation of responses against a putative native antigen structure on the surface. We subcloned the FSHR cDNA into a characterized expression vector (Figure 1B) and inoculated mice for the generation of antibody responses using direct plasmid injection followed by in vivo electroporation. Animals were immunized biweekly, and sera were collected a week after each immunization for analysis of antibody levels (Figure 1C).

To detect anti-FSHR antibodies that would bind to native FSHR expressed in the cell membrane, we stably transduced K562 cells to overexpress human FSHR (K562-FSHR). To validate the correct folding and functionality of the recombinant FSHR, we tested the response of K562-FSHR cells to follicle-stimulating hormone (FSH). As expected, K562-FSHR cells increased their production of cyclic AMP (cAMP) and ERK phosphorylation upon FSH stimulation, but no response was observed in the parental K562 (Figure 1, D and E). In a preliminary experiment, we observed that the anti-FSHR antibody D2AP11 partially blocked cAMP production by FSH stimulation of FSHR. We did not see any difference in cAMP production in K562-FSHR cells only in the presence of D2AP11 antibody without addition of FSH (Figure 1F), suggesting that additional studies in this area are important to understand the mechanism and to further confirm this observation (24, 25).

To monitor the ability of immune sera to bind FSHR, we combined K562 (GFP^–^) and K562-FSHR (GFP^+^) cells at equal ratios and added sera diluted up to 1:1,000, followed by anti-mouse IgG APC-conjugated secondary antibody (Supplemental Figure 1, A and B; supplemental material available online with this article; https://doi.org/10.1172/jci.insight.162553DS1) (26, 27), and determined the fold mean fluorescence intensity (MFI) of K562-FSHR compared with wild-type K562. When a 1:1,000 serum dilution exceeded 20-fold MFI, the last immunization was performed 3 weeks from the previous immunization by boosting with FSHR-overexpressing A20 cells. Boosted animals were sacrificed 4 days later for hybridoma generation as described (28, 29). Two weeks after the fusion, we screened supernatants from fifteen 96-well plates using flow cytometry to analyze the potential hybridomas (Supplemental Figure 1C). The top 20 clones based on fold MFI were expanded for further analysis (Supplemental Figure 1D). We downselected a highly potent clone, D2AP11 (fold MFI 42.2), based on high binding specificity.

**Figure 2:**
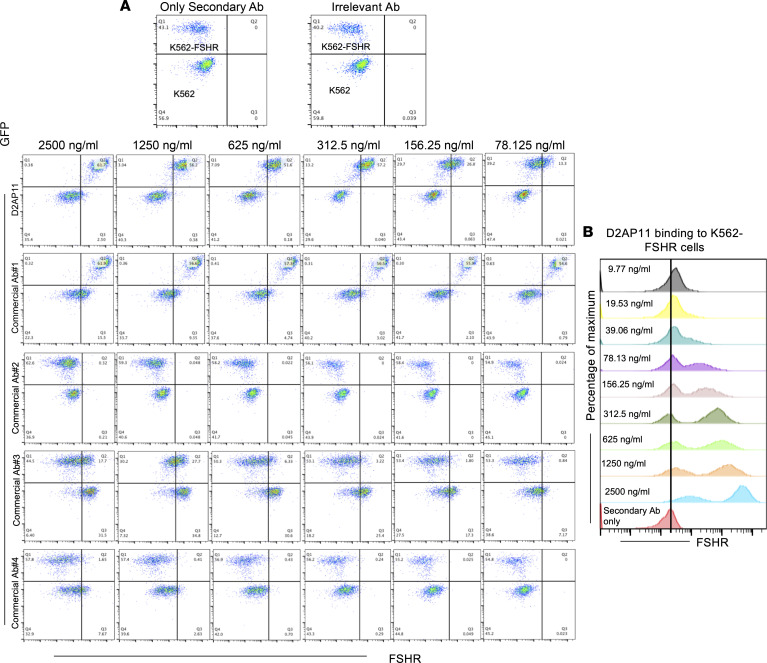
Binding potency of D2AP11 and commercial mouse anti–human FSHR antibodies. (**A**) Binding of D2AP11 anti-FSHR antibody and 4 different commercial antibodies (commercial Ab#1, #2, #3, and #4) in FSHR-overexpressing K562 cells analyzed by flow cytometry. Only secondary antibody control and irrelevant antibody control are shown at the top, where no binding to K562 and K562-FSHR cells was observed. The binding with different anti-FSHR antibodies was evaluated at different concentrations: 2,500 ng/mL, 1,250 ng/mL, 625 ng/mL, 312.5 ng/mL, 156.25 ng/mL, and 78.125 ng/mL. (**B**) Dose-dependent binding of D2AP11 anti-FSHR antibody to K562-FSHR cells analyzed by flow cytometry. D2AP11 binding was observed at a concentration as low as 9.77 ng/mL, indicating its potency.

We next compared the binding potential of this potent downselected antibody with those of 4 different commercially available mouse anti–human FSHR antibodies at different concentrations ranging from 2,500 ng/mL to 9.77 ng/mL. As shown in Figure 2A, D2AP11 exhibited high specific binding (K562-FSHR cells) and no nonspecific binding (K562 cells). Commercial Ab#1 showed high binding to K562-FSHR cells; however, at concentrations of 2,500, 1,250, and 625 ng/mL, it showed nonspecific binding to non-FSHR-expressing wild-type K562 cells. Commercial Ab#3 showed modest binding at 2,500 and 1,250 ng/mL; however, there was high nonspecific binding to K562 cells. We did not observe FSHR binding in the case of commercial Ab#2 and commercial Ab#4 when evaluated in different concentrations up to 2,500 ng/mL. Notably, D2AP11 mAb showed potent binding at a concentration as low as 9.77 ng/mL. The dose-dependent binding of this potent mAb clone is shown in Figure 2B and represents a new potent tool for human FSHR study.

### Anti-FSHR antibody binds FSHR with high specificity.

**Figure 3:**
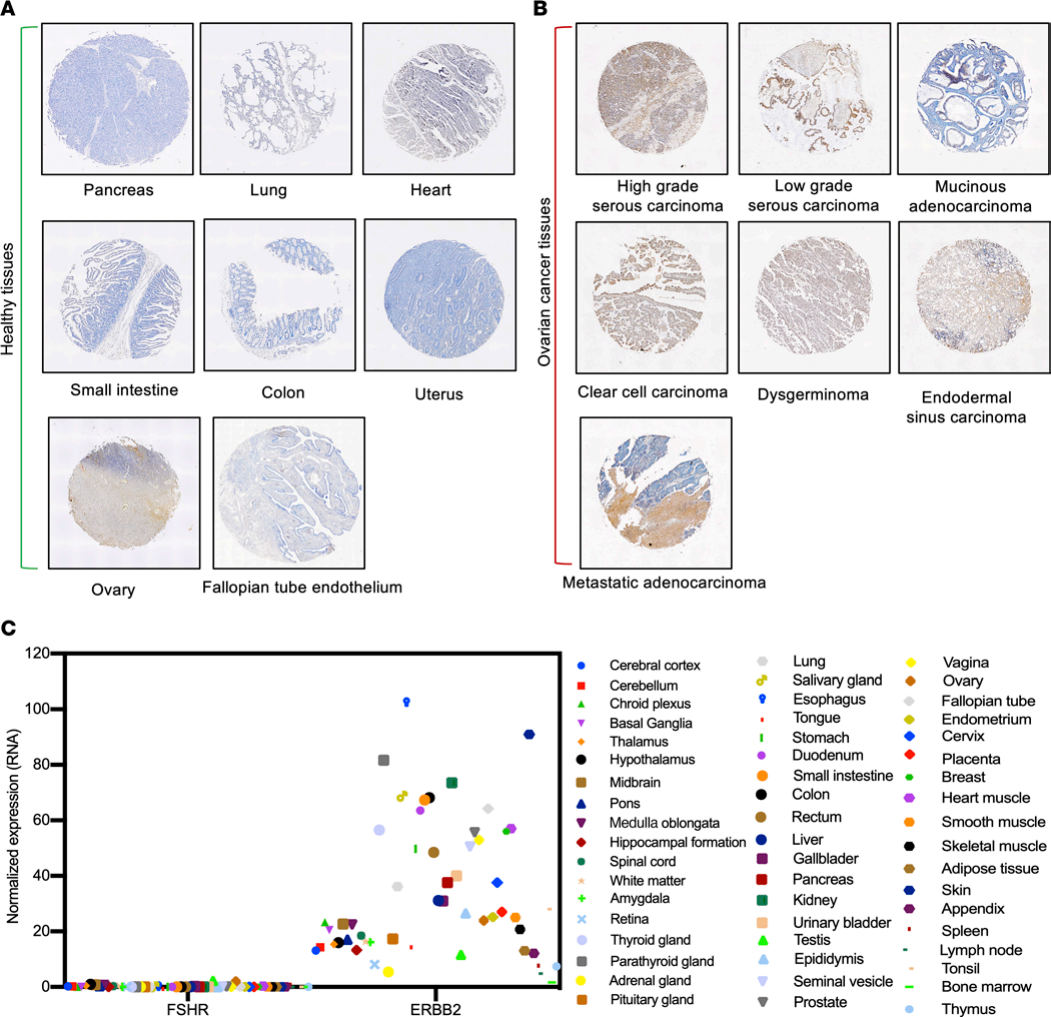
Binding of D2AP11 to healthy and OC tissues. (**A**) Representative images showing the binding of D2AP11 anti-FSHR antibody to healthy tissues of different human organs (pancreas, lung, heart, small intestine, colon, uterus, ovary, and fallopian tube endothelium), analyzed by immunohistochemistry. (**B**) Representative images showing the binding of D2AP11 anti-FSHR antibody to OC tissues of different pathological conditions (high- and low-grade serous carcinoma, mucinous adenocarcinoma, clear cell carcinoma, dysgerminoma, endodermal sinus carcinoma, and metastatic adenocarcinoma), analyzed by immunohistochemistry staining of OC TMA (US Biomax). The tissues (**A** and **B**) were viewed and imaged using Nikon NIS-Element Imaging system (×20, scale: 500 μm). Images were subjected to post-acquisition adjustments to optimize brightness, contrast, and image visibility. (**C**) Comparison of normalized RNA expression of FSHR and ERBB2/Her2 on 55 human tissue types, based on The Human Protein Atlas data.

**Figure 4:**
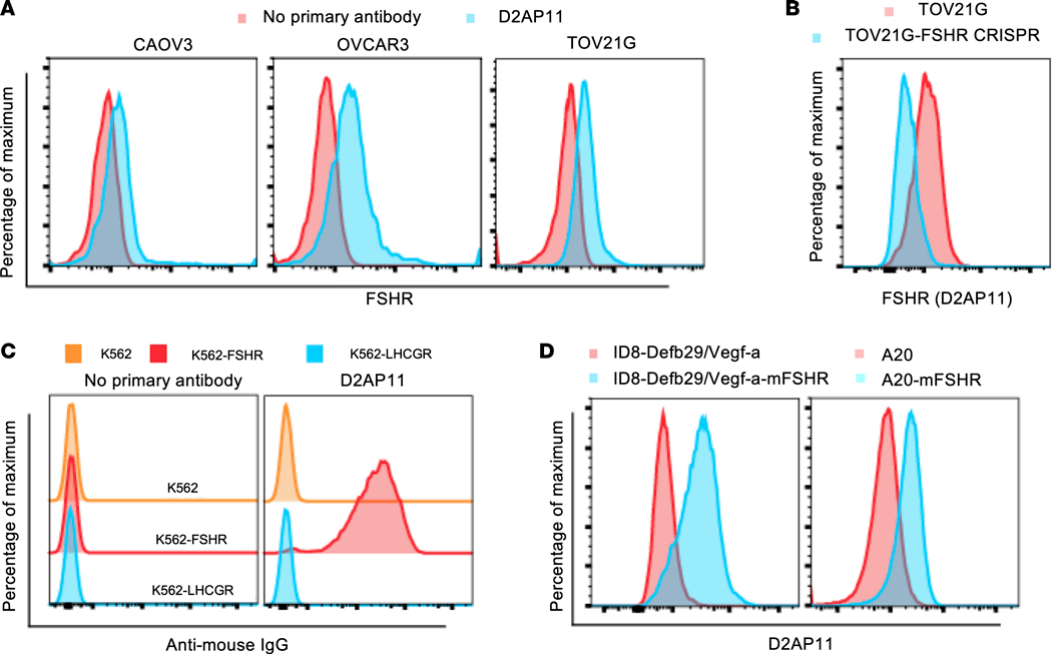
D2AP11 binds human and murine FSHR. (**A**) Flow cytometry plot of CAOV3, OVCAR3, and TOV21G cells stained with D2AP11 or no primary antibody followed by secondary APC-labeled antibody. (**B**) Flow cytometry plot of TOV21G cells, parental or after CRISPR-mediated deletion of FSHR, stained with D2AP11. (**C**) Flow cytometry plot of K562, K562-FSHR, and K562-LHCGR stained with D2AP11 or no primary antibody followed by secondary APC-labeled antibody. (**D**) Flow cytometry plot of A20 (GFP^–^)/A20-*Fshr* (GFP^+^) and ID8-*Defb29/Vegf-a* versus ID8-*Defb29/Vegf-a-Fshr* cells stained with D2AP11 (both cell lines were transfected with murine FSHR).

We characterized D2AP11 anti-FSHR antibody in detail for specificity by staining a panel of different healthy human tissues, including pancreas, lung, heart, small intestine, colon, uterus, ovary, and fallopian tube endothelium. For most of the healthy tissues, no significant binding of D2AP11 was observed. We observed D2AP11 binding in ovary and fallopian tube endothelium and high binding in different OC tissues (high-grade serous carcinoma, low-grade serous carcinoma, clear cell carcinoma, dysgerminoma, mucinous carcinoma, endodermal sinus carcinoma, and metastatic adenocarcinoma) (Figure 3, A and B). Further studies on additional healthy and OC tissues are important. Based on data from The Human Protein Atlas, we compared the differential RNA expression of FSHR versus ERBB2/Her2; mAbs and small-molecule inhibitors against ERBB2/Her2 have been developed, and currently clinical trials focusing on Her2-targeted bispecific immune cell engagers are ongoing (30, 31). These comparisons on 55 different human tissue types (31) likely support that FSHR deserves additional study for targeted OC immunotherapy (Figure 3C and Supplemental Figure 2). We then further characterized D2AP11 in detail for binding to different FSHR-expressing cells. D2AP11 binds to OC cell lines spontaneously expressing FSHR (7, 32). Cell lines (CAOV3, OVCAR3, and TOV21G) all showed the expected expression of FSHR by D2AP11 staining (Figure 4A). To further confirm that the signal elicited by D2AP11 corresponded to FSHR, we performed CRISPR-mediated deletion of FSHR in the TOV21G cell line. Flow cytometric staining with D2AP11 antibody showed absence of binding to the TOV21G cell line after CRISPR-mediated FSHR knockout (Figure 4B). This clone was highly specific for FSHR, as K562 cells transfected with LHCGR, the homologous protein to FSHR (sequence homology ~46% in the ECD and ~72% in the 7TMD) (33), did not show cross-reactivity by flow cytometry analysis (Figure 4C). We further tested whether D2AP11 was also able to bind murine FSHR, as mouse models of disease are important to study the in vivo efficacy and safety of new therapies. We expressed murine FSHR in mouse tumor lines A20 and ID8-Defb29/Vegf-a and again tested binding of D2AP11 to transfected and untransfected cells by flow cytometry. We found that D2AP11 successfully bound murine FSHR as it did human FSHR (Figure 4D).

### Anti-FSHR antibody for detection of FSHR^+^ tumor cells.

**Figure 5:**
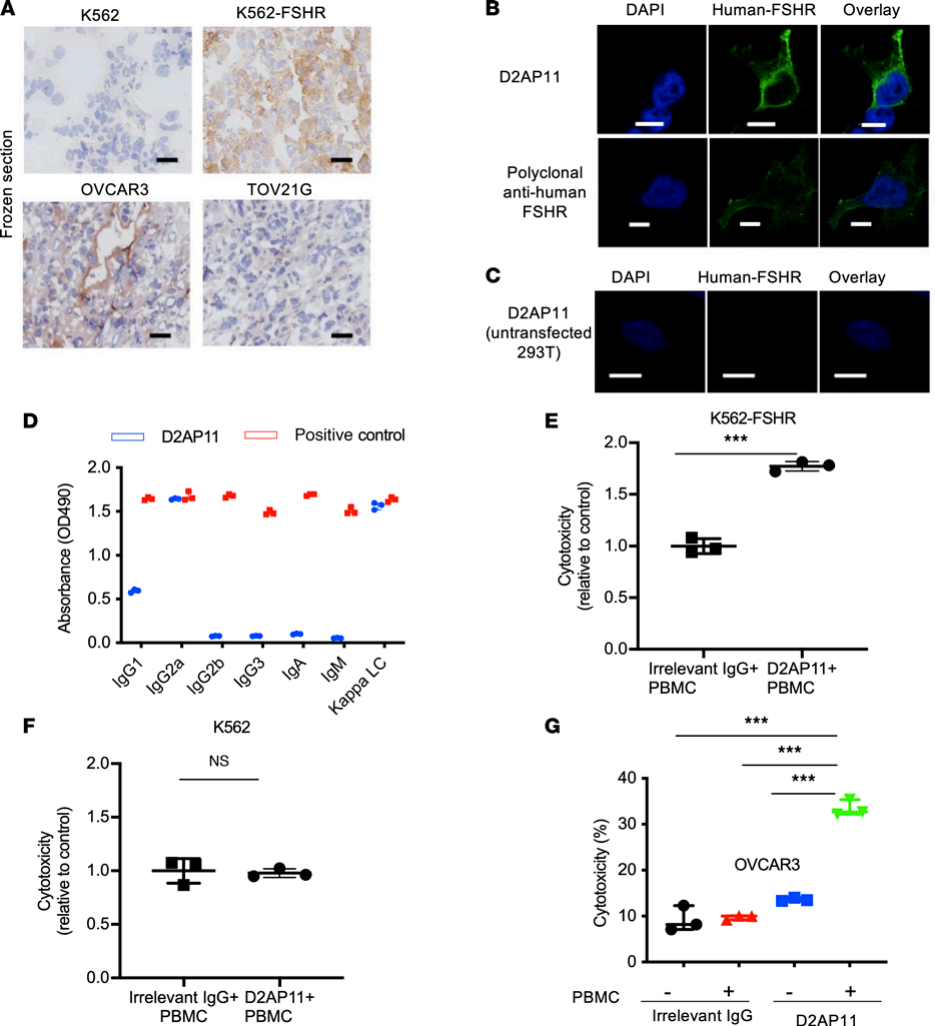
D2AP11 binds to FSHR in immunohistochemistry and immunocytochemistry and induces ADCC. (**A**) Immunohistochemistry images from frozen sections of tumors derived from K562, K562-FSHR, OVCAR3, and TOV21G cell lines stained with D2AP11. Original magnification, ×40; scale bars: 50 μm. (**B**) Immunofluorescence images of 293T cells transfected with human FSHR and stained with either mouse anti–human FSHR or D2AP11 antibodies followed by secondary anti-mouse IgG. (**C**) Immunofluorescence images of untransfected 293T cells stained with D2AP11 antibodies followed by secondary anti-mouse IgG. Scale bars: 10 μm (**B** and **C**). (**D**) Absorbance values of isotype ELISA performed on D2AP11 antibody. (**E**) Cytotoxicity mediated by antibody-dependent cell-mediated cytotoxicity (ADCC) of D2AP11 or irrelevant mouse IgG2a (C1.18.4) against K562-FSHR. (**F**) Cytotoxicity mediated by ADCC of D2AP11 or irrelevant mouse IgG2a (C1.18.4) against K562. (**G**) Cytotoxicity mediated by ADCC of D2AP11 or irrelevant mouse IgG2a (C1.18.4) against OVCAR3 cells. Error bars represent mean ± SEM; all the experiments were done in triplicate. *t* test and ANOVA. ***P < 0.001.

Immunohistochemical detection of proteins from biological samples is a common way of determining protein expression from tumors or other specimens to better classify them for prognostic or therapeutic purposes. To further explore whether D2AP11 detects FSHR^+^ tumor cells in immunohistochemistry, we generated solid tumors in NSG immunodeficient mice. To generate tumors, 5 million K562, K562-FSHR, OVCAR3, or TOV21G cells were injected in 50% PBS/Matrigel (Corning) into the axillary flank of NSG mice. D2AP11 detected FHSR from frozen tumor sections (Figure 5A). We stained human FSHR–transduced 293T cells with D2AP11. D2AP11 was able to bind human FSHR similarly to polyclonal anti-human, but not to untransfected 293T cells, confirming this activity (Figure 5, B and C).

### Anti-FSHR antibody induces antibody-dependent cell-mediated cytotoxicity.

To determine the isotype of D2AP11, we performed an ELISA and found D2AP11 to be IgG2a (Figure 5D), an isotype that can elicit antibody-dependent cell-mediated cytotoxicity (ADCC) (34). We first tested the ADCC capacity with K562 with or without FSHR. D2AP11 was able to increase the cytotoxic activity of PBMCs against K562-FSHR but not against K562 (Figure 5, E and F). To determine its ability to induce ADCC against unmodified FSHR^+^ OC cell lines, we cocultured OVCAR3 cells with PBMCs in the presence of D2AP11 or an irrelevant IgG2a antibody. We found that the physiological expression levels of FSHR in the OC cells were sufficient to be targeted by D2AP11-mediated cytotoxicity (Figure 5G) particularly with increasing doses of antibodies.

### Generation, expression, and binding of FSHR-targeted bispecific T cell engager.

**Figure 6:**
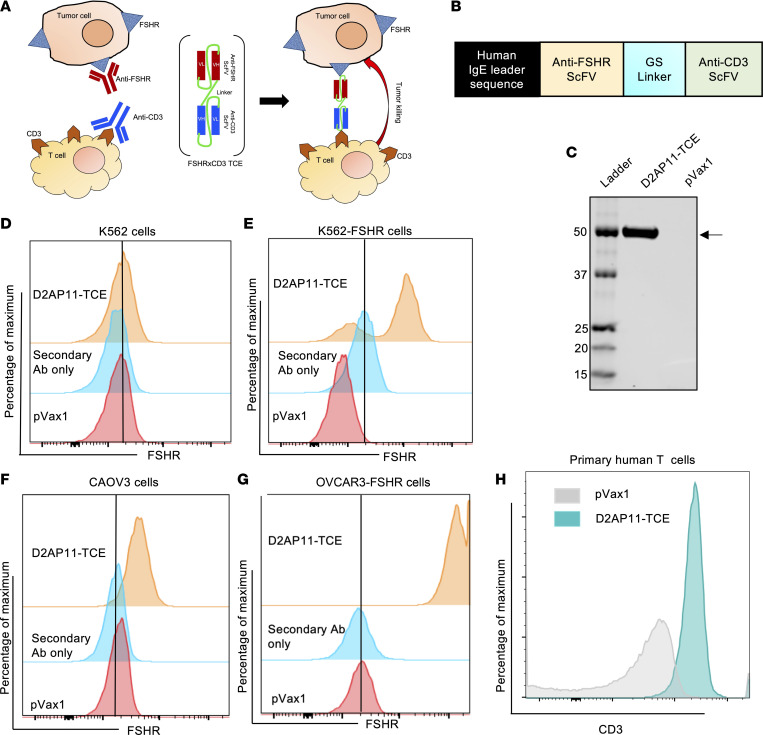
Generation, expression, and antitumor activity of D2AP11-TCE. (**A**) Cartoon of TCE engaging FSHR and the T cell receptor. VH, heavy chain variable region; VL, light chain variable region. (**B**) Schematic of DNA construct encoding D2AP11-TCE. GS, glycine-serine. (**C**) Western blot of in vitro expression of D2AP11-TCE or pVax1 empty vector after transfection in Expi293F cells. (**D**) The binding specificity of D2AP11-TCE was verified using K562 cells, which lack natural expression of FSHR. In K562 cells, no binding of D2AP11-TCE was observed. (**E**) Binding of D2AP11-TCE to FSHR-overexpressing K562 cells. (**F**) Binding of D2AP11-TCE to FSHR shown using the additional FSHR-expressing cell line CAOV3. The shift in the peak in D2AP11-TCE compared with pVax1 and secondary antibody alone indicates its binding to FSHR. (**G**) Binding of D2AP11-TCE to FSHR shown using OVCAR3 cells transduced with FSHR-encoding pBMN-I-GFP plasmid for overexpression of FSHR. There is a remarkable shift in the peak in FSHR-overexpressing OVCAR3 cells compared with empty vector and secondary antibody alone control. (**H**) Flow staining of primary human T cells with D2AP11-TCE and empty vector control; shift in peak denotes the binding of D2AP11-TCE to human T cells.

Bispecific T cell engagers (TCEs) represent a recent significant development in the field of monoclonal technology. As D2AP11 anti-FSHR antibody exhibited initial levels of ADCC, we sought to improve on this potential. We designed an FSHR targeting TCE (D2AP11-TCE) (35–37). We genetically optimized and fused the scFv of the FSHR mAb with the scFv of an optimized sequence we developed encoding anti-CD3 (modified from UCHT1) (Figure 6, A and B). D2AP11-TCE was efficiently expressed in vitro upon transfection of the DNA in Expi293F cells (Figure 6C). This bispecific showed no nonspecific binding to K562 cells, which do not have natural FSHR expression (Figure 6D), and retained binding to K562-FSHR cells (Figure 6E). Binding to FSHR was further confirmed in CAOV3 (Figure 6F) and OVCAR3-FSHR cells (Figure 6G). CD3 binding of D2AP11-TCE bispecific was confirmed using primary human T cells (Figure 6H).

### D2AP11-TCE induces potent killing in multiple ovarian tumor lines.

**Figure 7:**
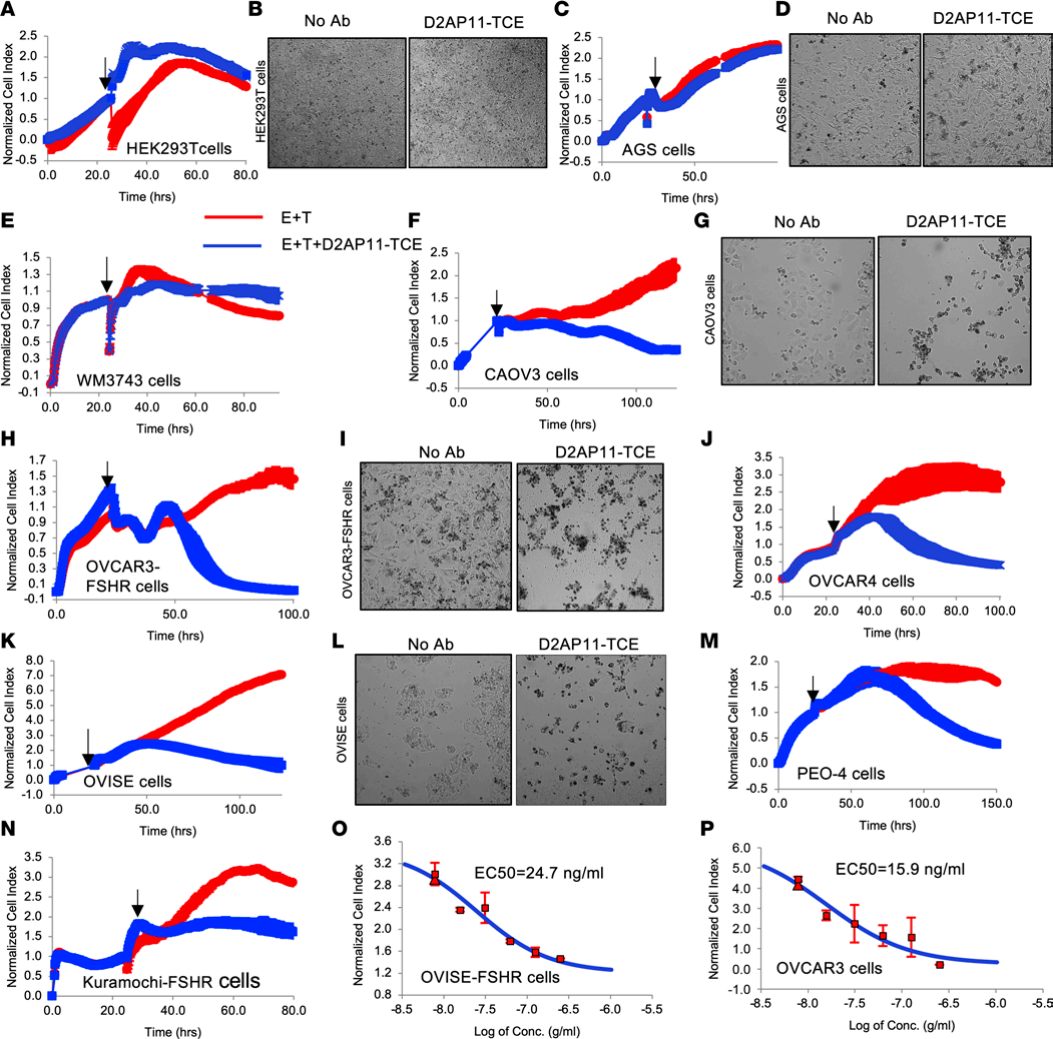
D2AP11-TCE induces specific killing of target OC cells. Assessment of the cytotoxic effect of D2AP11-TCE in FSHR-negative HEK293 cells (**A** and **B**), AGS gastric adenocarcinoma cells (**C** and **D**), and WM3743 human melanoma cells (**E**) as well as in the target human OC cell lines CAOV3 (**F** and **G**), OVCAR3-FSHR (**H** and **I**), OVCAR4 (**J**), OVISE (**K** and **L**), PEO-4 (**M**), and Kuramochi-FSHR (**N**) and dose-dependent killing of OVISE-FSHR (**O**) and OVCAR3 cells (**P**) in the presence of D2AP11-TCE and human PBMCs. In vitro cytotoxicity was measured based on impedance using xCELLigence real-time cell analyzer (RTCA) equipment (Agilent Technologies). The electrical conductivity was converted into the unitless cell index parameter by the xCELLigence device every 15 minutes, and images were captured at intervals of 1 hour. The data generated were normalized per the time point when the effector (E) cells (PBMCs) and D2AP11-TCE were added to the target (T) cells; E/T is 5:1 (**A**, **B**, **F**, **G**, **M**, and **N**) and 10:1 (**C**–**E**, **H**–**L**, **O**, and **P**). The data were analyzed using RTCA/RTCA Pro Software. No nonspecific killing was observed in HEK293T, AGS, and WM3743 cells, whereas potent killing was observed in CAOV3, OVCAR3-FSHR, OVCAR4, OVISE, PEO-4, and Kuramochi-FSHR target OC cells. Arrows indicate the time point at which D2AP11-TCE and effector cells were added to the target cells. Images shown display killing 2–3 days after the addition of effector cells and the TCE.

To determine the ability of the FSHR-targeted bispecific TCE, D2AP11-TCE, to induce cytotoxicity through activation of T cells, in vitro cytotoxicity assay was performed based on impedance using an xCELLigence real-time cell analyzer (RTCA). The target cells (CAOV3, OVCAR3-FSHR, OVCAR4, OVISE, PEO-4, and Kuramochi-FSHR) were placed in the xCELLigence RTCA device and incubated for 18–24 hours, and subsequently human PBMCs and D2AP11-TCE were added. As a control we used HEK293T cells, an FSHR-negative cell line (38). Notably, we did not observe off-target killing against FSHR-negative HEK293T cells (Figure 7, A and B). As additional controls, we used 2 other non-FSHR-expressing cells, AGS gastric adenocarcinoma (Figure 7, C and D) and WM3743 human melanoma (Figure 7E) cells, and D2AP11-TCE did not induce off-target toxicities in these 2 cells either. Evaluation of different FSHR-expressing ovarian tumor cells demonstrated that D2AP11-TCE was highly efficient in the killing of CAOV3 (Figure 7, F and G), OVCAR3-FSHR (Figure 7, H and I, and Supplemental Figure 3A), OVCAR4 (Figure 7J and Supplemental Figure 3, B and C), OVISE (Figure 7, K and L), PEO-4 (Figure 7M), and Kuramochi-FSHR cells (Figure 7N). We observed dose-dependent killing in OVISE-FSHR and OVCAR3 cells in the presence of D2AP11-TCE and human PBMCs/T cells with EC_50_ value of 24.7 ng/mL and 15.9 ng/mL, respectively (Figure 7, O and P, and Supplemental Figure 4). OVCAR4, a high-grade serous ovarian adenocarcinoma cell line, is reported to have distinct positive expression of the surface receptor; FSHR (39) and D2AP11-TCE induced potent killing in this cell line in the presence of human PBMCs as well as human T cells (Figure 7J and Supplemental Figure 3, B and C). The studied ovarian tumor lines harbor different cancer driver mutations and exhibit resistance to multiple anticancer drugs (Supplemental Table 1). Notably, Kuramochi and PEO-4 bear BRCA2 mutations, and the latter also exhibits resistance to PARP inhibitors (27, 40). In our assays there is no escape from D2AP11-TCE killing in these 2 resistant ovarian tumor lines as well. As shown in Figure 7, G, I, and L, 3 days after addition of effector cells and treatment with D2AP11-TCE, no attached tumor cells were observed in the treated wells. However, in control HEK293T and AGS cells (Figure 7, B and D), all cells were found to be growing healthily 2–3 days after addition of effector cells and D2AP11-TCE. Additionally, an irrelevant TCE targeting human IL-13 receptor α2 (IL13Rα2-TCE) did not exhibit toxicity in OVCAR3-FSHR cells in the presence of human PBMCs, indicating the role of the D2AP11 arm in specific killing of FSHR-expressing tumors (Supplemental Figure 5). Notably, D2AP11-TCE was able to induce significant toxicity to FSHR-positive OVCAR4 cells when compared with D2AP11 antibody, at concentrations around 1,000-fold lower, indicating the enhanced killing efficacy of D2AP11 through its design as a bispecific engager, D2AP11-TCE (Supplemental Figure 6, A and B). EC_50_ values of D2AP11 and D2AP11-TCE were obtained at 30.3 μg/mL and 11.3 ng/mL, respectively, indicating approximately 1,000-fold higher potency of D2AP11-TCE compared with the anti-FSHR antibody D2AP11 (Supplemental Figure 6, C and D).

### Cytokine/cytotoxic molecule secretion profile of FSHR-targeting TCE.

**Figure 8:**
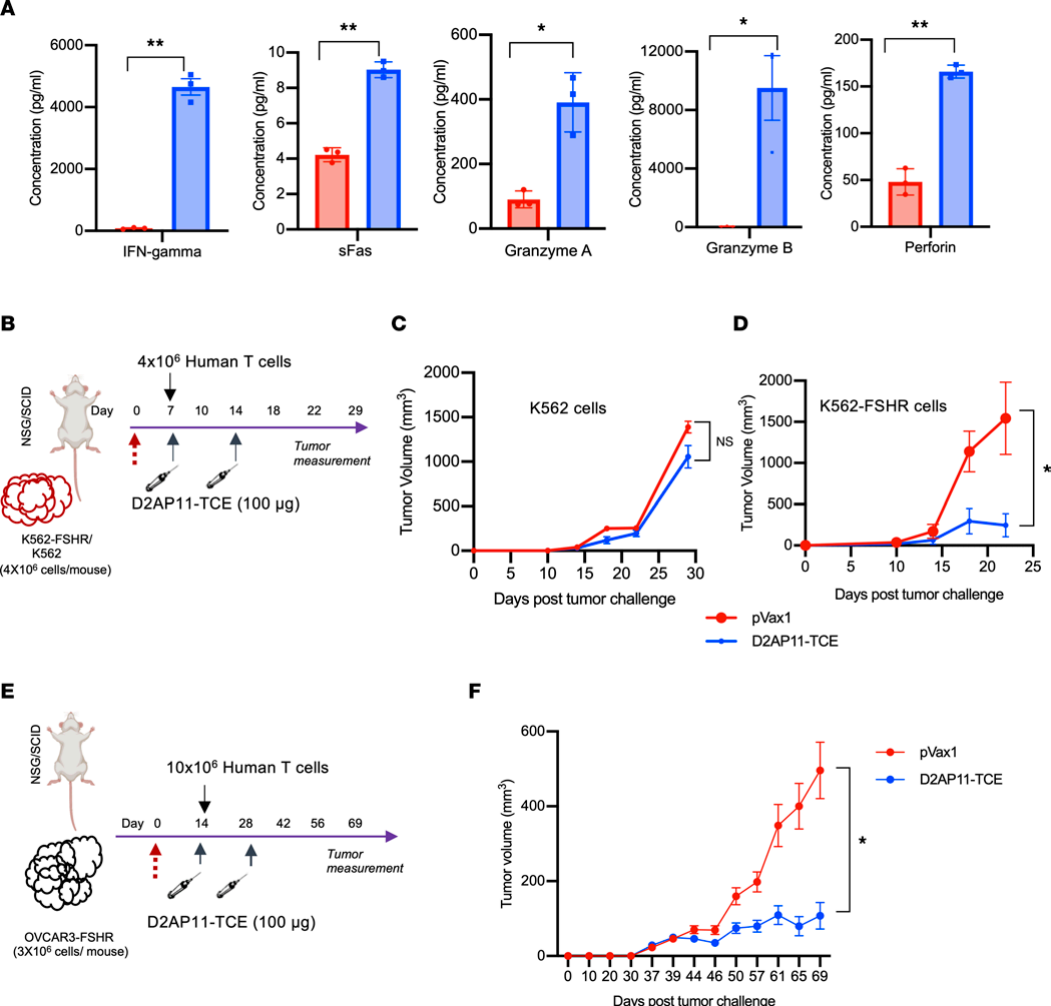
Cytokine/cytotoxic molecule secretion profile and in vivo activity of D2AP11-TCE. (**A**) Secretion profile of IFN-γ, sFas, granzymes A and B, and perforin in the presence of D2AP11-TCE upon coculturing of OVCAR3-FSHR and human PBMCs; E/T = 10:1. The supernatants analyzed were collected 48 hours after the addition of effector cells and TCE to target OVCAR3-FSHR cells. PBMCs from 3 different donors were used. Error bars represent mean ± SEM. *t* test. *P < 0.05, **P < 0.01. (**B**) Schematic of tumor study to evaluate the effect of D2AP11-TCE on tumor progression in K562/K562-FSHR–challenged NSG mouse model. (**C**) Average growth curve of K562 tumors grafted into NSG mice treated with D2AP11-TCE or empty vector (*n* = 5 mice per group). (**D**) Average growth curve of K562-FSHR tumors grafted into NSG mice treated with D2AP11-TCE or empty vector (*n* = 5 mice per group). (**E**) Schematic of tumor study to evaluate the effect of D2AP11-TCE on tumor progression in OVCAR3-FSHR–challenged NSG mouse model. (**F**) Average growth curve of OVCAR3-FSHR tumors grafted into NSG mice treated with D2AP11-TCE or empty vector (*n* = 10 mice per group). Two-way ANOVA. *P < 0.05.

Cytokines are involved in promoting the proliferation, survival, differentiation, and activation of lymphocytes (41). Different findings of bispecific TCEs as well as CARs suggest that cytokines secreted upon target cell ligation cause the lysis of antigen-negative tumor cells in close proximity to the antigen-specific engagement (42). We were interested in examining the cytokine/cytotoxic molecule secretion profile of D2AP11-TCE. Incubation of OVCAR3-FSHR target cells plus human PBMCs with D2AP11-TCE led to the significant induction of IFN-γ, soluble Fas (sFas), granzyme A, granzyme B, and perforin compared with empty vector control at 48 hours, and this construct drove robust tumor antigen-specific killing (Figure 8A). These effector molecules are known to possess potential to change the tumor microenvironment and to induce endogenous antitumor immunity (42).

### FSHR-targeted TCE decreases tumor burden in vivo.

To evaluate the in vivo antitumor effects of D2AP11-TCE, we used an in vivo challenge model we have developed. For this model we administered K562 cells or FSHR-overexpressing K562 cells (K562-FSHR) to NSG mice (Figure 8B). The mice were inoculated with DNA-encoded D2AP11-TCE (100 μg) and human T cells as described. Interestingly, there were tumor escapes in K562-challenged mice in both D2AP11-TCE–treated and empty vector control groups (Figure 8C), whereas there was a significant reduction in tumor volume in the D2AP11-TCE–treated group compared with empty vector control in the K562-FSHR–challenged mice (Figure 8D). After confirming the specificity and potency of D2AP11-TCE in this model, we further examined its effect in the OVCAR3-FSHR–challenged ovarian tumor mouse model. Fourteen days after tumor implantation, DNA-encoded D2AP11-TCE (100 μg) or empty vector (100 μg) was administered twice, 2 weeks apart, for in vivo antibody generation. On day 14, mice were inoculated with human T cells (10 million per mouse), and tumor volumes were measured periodically (Figure 8E). Treatment using this bispecific led to significantly decreased tumor burden in OVCAR3-FSHR tumor–bearing mice, while no similar impact was observed in the control group (Figure 8F), supporting the potential of this approach for therapeutic development against OC.

## Discussion

Despite important advances in the field of OC therapy, recurrent OC still presents a poor prognosis associated with a highly lethal cancer phenotype (1, 43, 44). Although there are multiple reasons to suppose that OC would respond favorably to treatment with immunotherapy, yet the immunotherapy response rates among OC patients remain fairly modest (45). A number of different immune targets have been used in the last few years for targeting OC through active (mesothelin, NY-ESO-1, p53, HER2/Neu, WT-1), passive (H7-B4, EpCAM, CA-125, CD-25, folate receptor α, PD-1/PD-L1, CTLA-4), and adoptive approaches (adoptive T cell therapy, naturally occurring T cell therapies, genetically modified T cell therapies, DC therapies) (46). Notably, a major obstacle in the development of targeted therapies is finding targets with specific expression confined to the surface of tumor cells, but not off-target tissues (7). FSHR is one such target with selective expression on ovarian granulosa cells (7), and thus we were moved to develop tools allowing for its consideration as a potential target in OC. FSH is a critical ovarian epithelial cell growth–inducing factor, which functions through binding to FSHR. Overexpression of FSHR is responsible for the upregulation of oncogenic pathways and increased proliferation of epithelial OC. Hence FSHR could be used as an important therapeutic target for directing T cells against OC (7). We have recently reported the potential for tumor impact, tolerability, and safety of targeting FSHR by vaccination using an immunocompetent mouse model (47). Injection of optimized DNA sequences followed by electroporation confers overexpression of the protein in its native conformation capable of eliciting potent cellular and humoral immune responses (48, 49). Here we extend this work using this approach for generation of anti-FSHR mAbs and study the resulting reagents as biologics. We describe the generation and characterization of a potent anti-FSHR antibody and study its application as a tool for immunotherapy of OC. We identified several clones, among them clone D2AP11, which was selected for additional study based on its consistent high cell-binding ability in flow analysis. The mAb supports detection of FSHR expression in samples by multiple methods, including flow cytometry, IFA, and immunohistochemistry. This suggests that additional study is warranted for this new reagent to extend its potential use in the clinic, where it may aid in determining the FSHR status of patient samples for personalized medicine approaches.

An important recent tool in the field of antibody technology for cancer therapy are bispecific TCE approaches (50). Bispecific TCEs can redirect both CD4^+^ as well as CD8^+^ T cells for the killing of tumor cells and are independent of intrinsic antigen-specific T cell receptor recognition by the T cells (13). While most of these are in preclinical and early clinical study, there are a very few approved cancer bispecific products. Blinatumomab (Blincyto) is used as a therapy for acute lymphoblastic leukemia (ALL) that targets CD19 on B cells and engages T cells through linked anti-CD3 binding (51); tebentafusp-tebn (Kimmtrak, Immunocore Ltd.), a bispecific gp100 peptide–HLA–directed CD3 TCE, was approved for patients with unresectable or metastatic uveal melanoma (30), supporting the importance of this approach. Blinatumomab displayed antileukemic function in adults with relapsed/refractory B precursor ALL depicted by negative prognostic factors. Most reported grade 3 or worse adverse events, including febrile neutropenia, neutropenia, and anemia. Patients displayed grade 3 cytokine release syndrome (2%) and neurologic events of the worst grades, grade 3 (11%) and grade 4 (2%) (52). Treatment with tebentafusp led to longer overall survival of metastatic uveal melanoma patients with no prior treatment received. The most frequent treatment-associated adverse events reported were cytokine-mediated events and skin-related events, including rash (83%), pyrexia (76%), and pruritus (69%). However, the incidence and severity of these events were reported to decrease after the first 3 or 4 doses (53). The benefit of these bispecific TCE approaches in severe disease appears to be of importance, and more studies can benefit the patients with severe disease.

Based on the specificity of the D2AP11 anti-FSHR clone, we reasoned this could be an important target for OC bispecific TCE approach. D2AP11-TCE was highly potent in tumor-specific cell killing, as evaluated against a panel of different human ovarian tumor cells (Figure 7). We included cell lines that display resistance against different chemotherapies, including HDAC inhibitors, Wee inhibitors, microtubule stabilizers, DNA alkylating agents, etc. (Supplemental Table 1) (26, 27). Nearly 23% of OC patients relapse within 6 months after their primary chemotherapy, with another 60% of patients reported to relapse after 6 months (54). Treatment escapes represent significant hurdles for OC therapeutic approaches. D2AP11-TCE showed significant killing against these different drug-resistant OC cell lines: OVISE, CAOV3, Kuramochi, OVCAR3, OVCAR4, and PEO-4. Germline mutations in BRCA1 or BRCA2 tumor suppressor genes, which play important roles in homologous recombination, are found in approximately 10% of cases of epithelial OC (55, 56). Although poly(ADP-ribose) polymerase inhibitors (PARPis) demonstrate impressive activity in sporadic high-grade serous OC as well as BRCA-related OC, unfortunately, as with classical chemotherapy, many patients are eventually reported to acquire resistance to PARPi treatment (4, 57–59), suggesting that the development of approaches targeting such OC tumors remains important. Interestingly, D2AP11-TCE was highly effective against cells harboring mutations in BRCA1 (OVISE) and BRCA2 (Kuramochi, PEO-4) and also gene signatures exhibiting resistance to PARPis (PEO-4) (Supplemental Table 1) (26, 27). Thus, D2AP11-TCE seems to have clinical benefit against a diverse panel of genetic and immune escape that has been previously documented in OC therapy. These studies provide a foundation supporting that D2AP11-TCE might have a value in combination with other therapeutic approaches, including chemotherapy, small molecules, and immunomodulatory drugs such as checkpoint inhibitors. However, future studies addressing this question are important.

We used direct DNA in vivo delivery to rapidly evaluate this new potential tool in OC challenge models. Notably, the TCE targeting FSHR was found to have high potency in attenuating tumor burden/tumor progression in vivo in an ovarian tumor–bearing mouse model. To the best of our knowledge, this is the first report of a FSHR-targeted bispecific TCE. PBMCs from 12 different healthy human donors were used across these studies. To our knowledge, this is also the first scFv-based therapeutic developed for FSHR. This could be useful for designing other scFv-based therapeutics such as CAR T cells targeting FSHR for OC.

These studies demonstrate, for the first time to our knowledge, the utility of targeting FSHR for a major subset of OC and highly immune-potent bispecific tools focused on FSHR and CD3, to impact tumor growth in vivo. Targeting of diverse populations of OC and the ability to complement currently available tools for treatment of this disease support study in additional FSHR-positive tumor models. Further studies are warranted for potential translational advancement of this tool for a variety of ovarian and other cancers expressing FSHR.

## Methods

### Cell lines and animals.

ID8-*Defb29/Vegf-a-Fshr*, ID8-*Defb29/Vegf-a*, OVCAR3, CAOV3, and TOV21G cells were provided by J.R. Conejo-Garcia (Department of Immunology, Moffitt Cancer Center, Tampa, Florida, USA). OVISE, OVCAR4, PEO-4, and Kuramochi cells were provided by R. Zhang (Immunology, Microenvironment and Metastasis Program, The Wistar Institute, Philadelphia, Pennsylvania, USA). WM3743 cells were provided by M. Herlyn (Molecular and Cellular Oncogenesis Program, The Wistar Institute). Human embryonic kidney 293T, Expi293F, gastric adenocarcinoma AGS, and murine myeloma cell line Sp2.0/0 were obtained from ATCC. OVCAR3, OVISE, and Kuramochi cells were retrovirally transduced with human FSHR as described previously (47). K562 and A20 were purchased from ATCC and retrovirally transduced to express human and murine FSHR, respectively. The expression vector pBMN-I-GFP (from G. Nolan Lab, Stanford University, Palo Alto, California, USA) was purchased from Addgene.

### Design of FSHRxCD3 TCE.

We designed FSHRxCD3 DNA-encoded bispecific TCE by encoding a codon-optimized scFv of FSHR mAb (D2AP11) followed by the scFv of a modified UCHT1 anti–human CD3 antibody with the addition of an IgE leader sequence. The construct was subcloned into a modified pVax1 expression vector (37). FSHRxCD3 TCE is designated as D2AP11-TCE.

### Flow cytometry.

We used a BD LSRII flow cytometer for staining of cells. A FACSAria cell sorter (BD Biosciences) was used for the sorting of cells stably expressing FSHR. Anti-human and anti-mouse antibodies used were directly fluorochrome conjugated. PE–secondary anti-human (H+L) (PA1-86078, Invitrogen), PE/AF647–secondary anti-human F(ab′)_2_ (109-605-097, Jackson ImmunoResearch Laboratories Inc.), and APC–secondary anti-mouse IgG (405308, BioLegend) were used. Live/Dead Violet viability kit (Invitrogen) was used to exclude dead cells from analysis.

### ELISA.

For isotyping D2AP11, we coated the ELISA plates with D2AP11 in PBS overnight. Then we blocked the plate and added the following HRP-conjugated antibodies: anti-mouse IgA (A90-103P, Bethyl), anti-mouse IgM (A90-101P, Bethyl), anti-mouse IgG1 (A90-105P, Bethyl), anti-mouse IgG2a (A90-107P, Bethyl), anti-mouse IgG2b (A90-109P, Bethyl), anti-mouse IgG3 (A90-111P, Bethyl), and anti-mouse κ light chain (A90-119P, Bethyl).

### Cyclic AMP determination.

We plated 25,000 K562 or K562-FSHR cells per well in 96-well plates. Cells were washed twice with warm PBS, then resuspended in 100 μL of serum-free RPMI with 0.5 mM IBMX (Cayman Chemical) with or without D2AP11 antibody. After incubation for 30 minutes at 37°C, we added FSH (50 ng/mL or 1 μg/mL) or PBS. One hour later we washed the cells with ice-cold PBS, lysed them, and performed cyclic AMP determination according to the manufacturer’s instructions (Cell Signaling).

### Immunoblotting.

Protein extraction, denaturation, and Western blotting were performed as previously described (29, 60, 61). Membranes were blotted with anti–phospho-p44/42 MAPK (Erk1/2) (Thr202/Tyr204) (9101, Cell Signaling) and anti–p44/42 MAPK (Erk1/2) (4695, Cell Signaling). Images were captured with ImageQuantLAS 4000 (GE Healthcare Life Sciences). For detection of D2AP11-TCE expression, goat anti-human IgG F(ab′)_2_ (109-005-006, Jackson ImmunoResearch Laboratories Inc.) and donkey anti-goat secondary antibodies (926-32214, LI-COR) were used, and membranes were scanned using a LI-COR Odyssey CLx imager.

### In vitro cytotoxicity analysis through measurement of luciferase expression.

We plated 10,000 OVCAR3 cells per well in a 96-well plate and, after 18 hours, added primary PBMCs. After a 4-hour coincubation, we stained the cells with 7AAD (Invitrogen), annexin V (BioLegend), and anti–human CD45 (304002, BioLegend), then performed a flow cytometry–based cytotoxicity assay as described previously (7). We stably transfected K562 and K562-FSHR with firefly luciferase. We plated 20,000 K562 and K562-FSHR cells expressing luciferase in a 96-well plate and coincubated them for 5 hours with PBMCs. After the incubation, we lysed the cells and measured luciferase expression using CytoTox Glo (Promega) as previously described (32). Cytotoxicity was calculated as (maximum viability control – individual well)/(maximum viability control – maximum death control) × 100 as a percentage or relative to the control (PBMCs with mouse IgG2a isotype control C1.18.4).

### In vitro cytotoxicity analysis using xCELLigence real-time cell analyzer.

In vitro cytotoxicity assay was performed based on impedance using xCELLigence real-time cell analyzer (RTCA) equipment (Agilent Technologies). The impedance is expressed in cell index (62). Target cells were seeded into disposable sterile 96-well E-plates of the xCELLigence RTCA device at final cell concentration of 1 × 10^4^ to 2 × 10^4^ cells per well. The instrument was placed in a CO_2_ incubator during the experiment and controlled by a cable connected to the control unit. The 96-well E-plate was placed in the xCELLigence RTCA device and incubated for 18–24 hours. Subsequently, the effector cells (human PBMCs or T cells; effector/target ratio = 5:1 and 10:1) and treatments were added. Real-time analysis was performed for 3–7 days. The electrical conductivity was converted into the unitless cell index parameter by the xCELLigence device every 15 minutes, and images were captured at 1-hour intervals. The data generated were normalized according to the time point when the effector cells and TCE were added to the target cells and were analyzed using RTCA/RTCA Pro Software. Human PBMCs and T cells from healthy donors were provided by the Human Immunology Core of the University of Pennsylvania (Philadelphia, Pennsylvania, USA).

### Immunohistochemistry and immunocytochemistry.

Mouse tumors were frozen in OCT (TissueTek) and frozen sections cut. HEK293T cells were grown on top of poly-l-lysine–coated cover slides (MilliporeSigma) and transfected using a human or murine FSHR expression vector. Slides were then fixed with 4% paraformaldehyde and permeabilized with 0.5% Triton X-100 in PBS. Sections were blocked using 5% normal goat serum followed by staining with D2AP11 antibody, then AF647-conjugated secondary antibodies specific for human (A-21445, Invitrogen) or mouse (A-21235, Invitrogen) IgG. Slides were viewed using a Leica TCS SP-5 confocal microscope and Leica LAS-X software (immunocytochemistry) or a Nikon ECLIPSE 80i microscope and NIS-Element Imaging (immunohistochemistry). For immunohistochemistry analysis of tissue microarray (TMA) slides (US Biomax), they were deparaffinized and rehydrated, followed by antigen retrieval, blocking with 5% normal goat serum, and staining with D2AP11 antibody and then biotinylated anti-mouse secondary antibody (BA-9200, Vector Laboratories). Then the TMA slides were incubated with peroxidase solution (Vector Laboratories) followed by DAB substrate and counterstained with hematoxylin (Leica). The slides were viewed and imaged using a Nikon NIS-Element Imaging system (×20, scale: 500 μm).

### Cytokine/cytotoxic molecule secretion profile analysis.

OVCAR3-FSHR (target) cells were plated at a density of 1 × 10^4^ cells per well. After overnight incubation, PBMCs (effector cells; effector/target = 10:1) and treatments (D2AP11-TCE and pVax1) were added to the target cells. After 48 hours, the supernatants were collected and analyzed for secreted proteins by LEGENDplex Human CD8/NK Panel multiplex bead-based assay (BioLegend) per the manufacturer’s protocol.

### Tumor challenges.

We challenged NOD/SCID-γ (NSG) mice with K562 and K562-FSHR cells. NSG mice were injected with 4 × 10^6^ K562 or K562-FSHR cells on the right flank subcutaneously. Seven days after the tumor became palpable, mice were inoculated with pVax1 (100 μg) or D2AP11-TCE (100 μg) followed by electroporation. The same day expression vector was given, we injected 4 × 10^6^ human T cells intraperitoneally into each mouse. The mice were inoculated with DNA twice, 1 week apart. For the OVCAR3-FSHR–challenged mouse model, mice were inoculated with 3 × 10^6^ OVCAR3-FSHR cells on the right flank subcutaneously. Fourteen days after the tumor became palpable, mice were inoculated with pVax1 (100 μg) or D2AP11-TCE (100 μg). The same day, we injected 10 × 10^6^ human T cells intraperitoneally into each mouse. The mice were inoculated with pVax1 or D2AP11-TCE twice, 2 weeks apart. Tumor sizes were monitored periodically via caliper measurements. Mice were euthanized upon developing signs of graft-versus-host disease. Tumor volume (*V*) was calculated per the formula *V* = (length × width^2^)/2; width is the side with smaller measurement. Human T cells from healthy donors were provided by the Human Immunology Core of the University of Pennsylvania.

### Statistics.

All statistical analyses were done using GraphPad Prism. *P* values less than 0.05 were considered statistically significant. Differences between the means of experimental groups were calculated using a 2-tailed unpaired Student’s *t* test or 1-way ANOVA where more than 2 quantitative variables were measured. Error bars represent SEM. Comparisons between tumor size at each time point were done using 2-way ANOVA with Fisher’s least significant difference test.

### Study approval.

Animal experiments were approved by the Institutional Animal Care and Use Committee at The Wistar Institute.

## Author contributions

DB, PSB, APP, and DBW conceptualized the study. DB, APP, PSB, AJK, DWK, RZ, and DBW provided methodology. DB, PSB, APP, and DBW provided investigation. DB, APP, AJK, PSB, XZ, KL, RPO, and DHP acquired data. DB, APP, PSB, DWK, RZ, and DBW provided analysis or interpreted data. DB and APP wrote the original draft of the manuscript. DB, PSB, APP, AJK, XZ, KL, RPO, DHP, DWK, RZ, and DBW reviewed and edited the manuscript.

## Supplementary Material

Supplemental data

## Version 1 11/22/2022

Electronic publication

## References

[1] Kurnit KC (2021). Updates and new options in advanced epithelial ovarian cancer treatment. Obstet Gynecol.

[2] https://www.cancer.org/cancer/ovarian-cancer/about/key-statistics.html.

[3] Barnes BM (2021). Distinct transcriptional programs stratify ovarian cancer cell lines into the five major histological subtypes. Genome Med.

[4] Yang C (2020). Immunotherapy for ovarian cancer: adjuvant, combination, and neoadjuvant. Front Immunol.

[5] Banno K (2014). Application of microRNA in diagnosis and treatment of ovarian cancer. Biomed Res Int.

[7] Perales-Puchalt A (2017). Follicle-stimulating hormone receptor is expressed by most ovarian cancer subtypes and is a safe and effective immunotherapeutic target. Clin Cancer Res.

[8] Hayes DF (2007). HER2 and response to paclitaxel in node-positive breast cancer. N Engl J Med.

[9] Pegram MD (1998). Phase II study of receptor-enhanced chemosensitivity using recombinant humanized anti-p185HER2/neu monoclonal antibody plus cisplatin in patients with HER2/neu-overexpressing metastatic breast cancer refractory to chemotherapy treatment. J Clin Oncol.

[10] Maloney DG (1997). IDEC-C2B8 (Rituximab) anti-CD20 monoclonal antibody therapy in patients with relapsed low-grade non-Hodgkin’s lymphoma. Blood.

[11] Bast RC (1983). A radioimmunoassay using a monoclonal antibody to monitor the course of epithelial ovarian cancer. N Engl J Med.

[12] Siddall JK (1986). Measurements of serum gamma-seminoprotein and prostate specific antigen evaluated for monitoring carcinoma of the prostate. Clin Chem.

[13] Dao T (2015). Therapeutic bispecific T-cell engager antibody targeting the intracellular oncoprotein WT1. Nat Biotechnol.

[14] Aldoss I (2017). Redirecting T cells to eradicate B-cell acute lymphoblastic leukemia: bispecific T-cell engagers and chimeric antigen receptors. Leukemia.

[15] Gedeon PC (2013). An EGFRvIII-targeted bispecific T-cell engager overcomes limitations of the standard of care for glioblastoma. Expert Rev Clin Pharmacol.

[16] Schlereth B (2005). Eradication of tumors from a human colon cancer cell line and from ovarian cancer metastases in immunodeficient mice by a single-chain Ep-CAM-/CD3-bispecific antibody construct. Cancer Res.

[17] Crawford A (2019). A Mucin 16 bispecific T cell-engaging antibody for the treatment of ovarian cancer. Sci Transl Med.

[18] Bargou R (2008). Tumor regression in cancer patients by very low doses of a T cell-engaging antibody. Science.

[19] Hipp S (2017). A novel BCMA/CD3 bispecific T-cell engager for the treatment of multiple myeloma induces selective lysis in vitro and in vivo. Leukemia.

[20] Baeuerle PA, Reinhardt C (2009). Bispecific T-cell engaging antibodies for cancer therapy. Cancer Res.

[21] Mariani S (2006). Expression and cellular localization of follicle-stimulating hormone receptor in normal human prostate, benign prostatic hyperplasia and prostate cancer. J Urol.

[22] Radu A (2010). Expression of follicle-stimulating hormone receptor in tumor blood vessels. N Engl J Med.

[23] Fan QR, Hendrickson WA (2005). Structure of human follicle-stimulating hormone in complex with its receptor. Nature.

[24] Zhu D (2018). Extragonadal effects of follicle-stimulating hormone on osteoporosis and cardiovascular disease in women during menopausal transition. Trends Endocrinol Metab.

[25] Haldar S (2022). Overview of follicle stimulating hormone and its receptors in reproduction and in stem cells and cancer stem cells. Int J Biol Sci.

[26] https://cellmodelpassports.sanger.ac.uk/.

[27] Sakai W (2009). Functional restoration of BRCA2 protein by secondary BRCA2 mutations in BRCA2-mutated ovarian carcinoma. Cancer Res.

[28] Choi H (2020). Synthetic nucleic acid antibody prophylaxis confers rapid and durable protective immunity against Zika virus challenge. Hum Vaccin Immunother.

[29] Bordoloi D (2021). Identification of novel neutralizing monoclonal antibodies against SARS-CoV-2 spike glycoprotein. ACS Pharmacol Transl Sci.

[30] https://www.fda.gov/drugs/resources-information-approved-drugs/fda-approves-tebentafusp-tebn-unresectable-or-metastatic-uveal-melanoma.

[31] https://www.proteinatlas.org/.

[32] Zhang XY (2009). Follicle-stimulating hormone peptide can facilitate paclitaxel nanoparticles to target ovarian carcinoma in vivo. Cancer Res.

[33] Ulloa-Aguirre A (2018). Structure-function relationships of the follicle-stimulating hormone receptor. Front Endocrinol (Lausanne).

[34] Akiyama Y (1984). Induction of mouse IgG2a- and IgG3-dependent cellular cytotoxicity in human monocytic cells (U937) by immune interferon. Cancer Res.

[35] Gary EN (2021). A novel mouse AAV6 hACE2 transduction model of wild-type SARS-CoV-2 infection studied using synDNA immunogens. iScience.

[36] Patel A (2018). In vivo delivery of synthetic human dna-encoded monoclonal antibodies protect against ebolavirus infection in a mouse model. Cell Rep.

[37] Perales-Puchalt A (2019). DNA-encoded bispecific T cell engagers and antibodies present long-term antitumor activity. JCI Insight.

[38] Urbanska K (2015). Follicle-stimulating hormone receptor as a target in the redirected T-cell therapy for cancer. Cancer Immunol Res.

[39] https://depmap.org/portal/.

[40] He YJ (2018). DYNLL1 binds to MRE11 to limit DNA end resection in BRCA1-deficient cells. Nature.

[41] Romain G (2014). Antibody Fc engineering improves frequency and promotes kinetic boosting of serial killing mediated by NK cells. Blood.

[42] Slaney CY (2018). CARs versus BiTEs: a comparison between T cell-redirection strategies for cancer treatment. Cancer Discov.

[43] Izar B (2020). A single-cell landscape of high-grade serous ovarian cancer. Nat Med.

[44] Hamanishi J (2016). Immune checkpoint inhibition in ovarian cancer. Int Immunol.

[45] Coleman RL (2016). Ovarian cancer in 2015: insights into strategies for optimizing ovarian cancer care. Nat Rev Clin Oncol.

[46] Schwab CL (2014). Past, present and future targets for immunotherapy in ovarian cancer. Immunotherapy.

[47] Perales-Puchalt A (2019). Engineered DNA vaccination against follicle-stimulating hormone receptor delays ovarian cancer progression in animal models. Mol Ther.

[48] Tebas P Safety and immunogenicity of an anti–Zika virus DNA vaccine — preliminary report. N Engl J Med.

[49] Yan J (2013). Highly optimized DNA vaccine targeting human telomerase reverse transcriptase stimulates potent antitumor immunity. Cancer Immunol Res.

[50] Bhojnagarwala PS (2022). In vivo DNA launched bispecific T cell engager (dBTE) targeting IL13Rα2 controls tumor growth in an animal model of glioblastoma multiforme. Mol Ther Oncolytics.

[51] Sheridan C (2021). Bispecific antibodies poised to deliver wave of cancer therapies. Nat Biotechnol.

[52] Topp MS (2015). Safety and activity of blinatumomab for adult patients with relapsed or refractory B-precursor acute lymphoblastic leukaemia: a multicentre, single-arm, phase 2 study. Lancet Oncol.

[53] Nathan P (2021). Overall survival benefit with tebentafusp in metastatic uveal melanoma. N Engl J Med.

[54] Pignata S (2017). Treatment of recurrent ovarian cancer. Ann Oncol.

[55] Safra T (2011). BRCA mutation status and determinant of outcome in women with recurrent epithelial ovarian cancer treated with pegylated liposomal doxorubicin. Mol Cancer Ther.

[56] Hennessy BT (2009). Ovarian cancer. Lancet.

[57] McMullen M (2020). Overcoming platinum and PARP-inhibitor resistance in ovarian cancer. Cancers (Basel).

[58] Franzese E (2019). PARP inhibitors in ovarian cancer. Cancer Treat Rev.

[59] Liu JF (2014). PARP inhibitors in ovarian cancer: current status and future promise. Gynecol Oncol.

[60] Tesone AJ (2016). Satb1 overexpression drives tumor-promoting activities in cancer-associated dendritic cells. Cell Rep.

[61] Bordoloi D (2021). Immunotherapy of prostate cancer using novel synthetic DNA vaccines targeting multiple tumor antigens. Genes Cancer.

[62] Durdagi S (2021). The neutralization effect of montelukaston SARS-CoV-2 is shown by multiscale in silicosimulations and combined in vitro studies. Mol Ther.

