# Supplementary material for: A mAb against surface-expressed FSHR engineered to engage adaptive immunity for ovarian cancer immunotherapy
Source: JCI Insight. 2022 Nov 22;7(22):e162553. doi: 10.1172/jci.insight.162553 (PMC9746812; doi:10.1172/jci.insight.162553)
Supplement: Supplemental data [file jciinsight-7-162553-s138.pdf]

Supplementary Table 1: Different ovarian tumor lines used in the study, types, cancer driver mutations, and drug resistance

Name	Type	Cancer driver mutations	Resistant drugs (*Drug targets*)	References
OVISE	Ovarian clear cell adenocarcinoma	ARID1A, ARID1B, BRCA1, CALR, ARHGAP35, SDHA, DDX5, ETV4, HSP90AB1, NFKBIE, FGFR4, LYL1, MDM2, PIK3CA, PPP2R1A, CD79B, PRKACA, PTPRB, RGL3, STAT3, UGT2B17, WNK4, STAT5B, TGIF1, ZNF429, ZNF780A,	FEN1_3940(*FEN1*), Tanespimycin (*HSP90*)	26
CaOV3	High grade ovarian serous adenocarcinoma	ASPSCR1, HSP90AB1, AMER1, ATR, BCL6, BCORL1, CAMTA1, BCR, CBLB, CCND2, CHD4, CHEK2, COL1A1, CR1, CRLF2, DGCR8, EIF4A2, ERC1, ETV5, FGFR3, HLA-B, KLHL6, KMT2A, LPP, MAPK1, MB21D2, MN1, NFKBIE, TBL1XR1, NSD2, NT5C3A, P2RY8, PAK2, PDPR, PIK3CB, PMS2, POLQ, POU5F1, PTK6, PTPN6, RASA2, RSPH10B2, SDHA, SFRP4, SMARCB1, CTLCL1, SOX2, SPOP, STK11, SUSD2, TERT, TP53, TP63, ZBTB20, ZNF-384, CCND3, MAP3K13, ZNF148,	Dacinostat (*HDAC*)	26
Kuramochi	High grade ovarian serous adenocarcinoma	ABI1, AKT2, ARHGAP35, BCL3, BRCA2, CBLC, CCNE1, CD79A, CDH10, CEBP1, CHCHD7, CIC, DEK, EIF3E, ERCC2, EXT1, FAM135B, GNAS, H4C9, HEY1, HLA-B, IL7R, KMT2B, KMT2C, KRAS, LATS2, LIFR, MYC, NBN, NCOA2, NDRG1, NEKBIA, NFATC2, NIPBL, PLAG1, POU2F2, PREX2, PRKD2, PTK6, RAD21, RECQL4, RHPN2, RSPH10B2, SALL4, SDC4, SDHA, SS18L1, TERT, TP53, UBR5, UGT2B17, ZMYM2	Dactolisib (*PI3K -class* 1, *MTORC1, MTORC2*), Wee1 inhibitor (*WEE1, CHEK*1), Buparlisib (*PI3Kalpha, PI3Kdelta, PI3Kbeta, PI3Kgamma*), UMI-77 (*MCL1*), Docetaxel (*Microtubule stabilizer*), AZD5438 (*CDK2*), Gallibiscoquinazole, Mitoxantrone, Irinotecan (*TOP1*), Entinostat (*HDAC1, HDAC*3), Oxaliplatin (*DNA alkylating agent*)	26

OVCAR3	High grade ovarian serous adenocarcinoma	AKT2, CAMTA1, CASZ1, CEBPA, CN0T3, DDX5, EFTUD2, ELK4, EPHA2, ETV4, KDM5C, KMT2B, MTOR, NPEPPS, PCM1CCNE1, PRDM16, PRDM2, RHPN2, RPL22, SLC45A3, SMC1A, SPEN, TNFRSF14, TP53, ZCRB1, ZNF331, ZNF721, ZNF780A, ZXDB	Sepantronium bromide (*BIRC5*), Foretinib (*MET, KDR, TIE2, VEGFR3/FLT4, RON, PDGFR, FGFR1, EGFR*), Belinostat (*HDAC1*), CAY10603 (*HDAC1, HDAC6*), AR-42 (*HDAC1*), CUDC-101 (*HDAC1-10, EGFR, ERBB2*), JW-7-24-1 (*LCK*), Omipalisib (*PI3K-class 1, MTORC1, MTORC2*), QL-XII-47 (*BTK, BMX*), THZ-2-102-1 (*CDK7*), AT-7519 (*CDK1, CDK2, CDK4, CDK6, CDK9*), UNC0638 (*G9a and GLP methyltransferases*), QL-X-138 (*BTK*), LDN-193189 (*BMP*), KIN001-244 (*PDK1*), OSI-027 (*MTORC1, MTORC2*), WZ3105 (*SRC, ROCK2, NTRK2, FLT3, IRAK1, others*), WZ3105 (*SRC, ROCK2, NTRK2, FLT3, IRAK1, others*)	26
OVCAR4	High Grade Ovarian Serous Adenocarcinoma	BRAF, BREB3L2, CAMTA1, CCNE1, CEBPA, CNOT3, CUX1, EZH2, FAM135B, KEL, KLK2, KMT2C, MET, MYC, NDRG1, PEG3, POLD1, POT1, PPP2R1A, RECQL4, RHPN2, SMO, SND1, TP53, TRIM24, TRRAP, U2AF2, UGT2B17, ZNF331	Pictilisib (PI3K-class 1), Eg5_9814 (KSP11), Taselisib (PI3K- beta sparing), Mirin (MRE11), AZD2014 (mTORC1, mTORC2), AZ960 (JAK2, JAK3), Teniposide, CDK9_5576 (CDK9), AZD5363 (AKT1, AKT2, AKT3, ROCK2), CDK9_5038 (CDK9)	26
PEO-4	Ovarian cystadenocarcinoma	BRCA2	Cisplatin (*DNA alkylating agent*), PARP inhibitor (*PARP*)	26, 27

Supp Figure 1

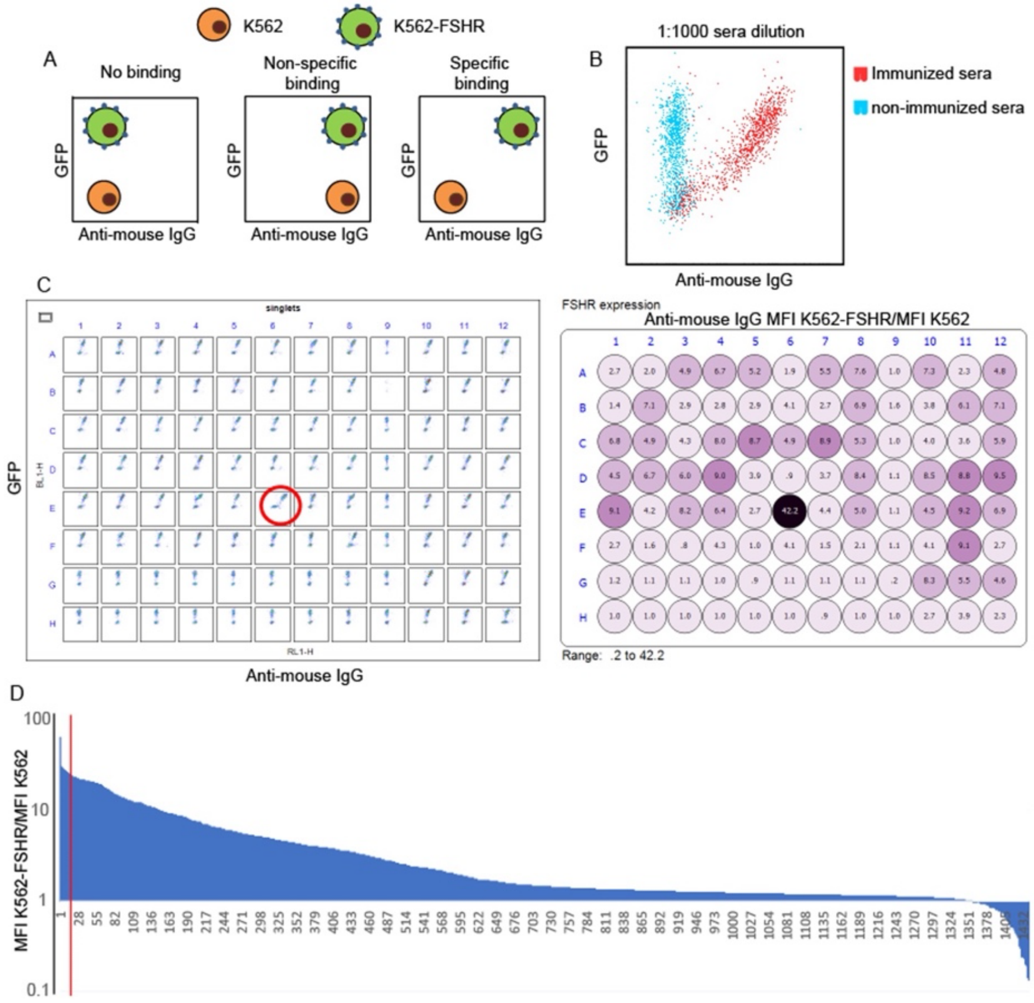

Supplementary Figure 1.

Screening of anti-human FSHR antibodies. (**A**) Scheme of flow cytometry plots representing the potential outcomes in the screening process with K562 and K562-FSHR. (**B**) Flow cytometry plot of K562 (GFP-)/K562-FSHR (GFP+) cells stained with sera from mice immunized with human FSHR or empty vector at 1:1000 dilution and anti-mouse IgG APC. (**C**) Representative of flow cytometric screening output strategy for detection of FSHR binding antibodies from hybridomas as flow plot and fold mean fluorescent intensity of K562-FSHR/K562 (**D**) Waterfall plot depicting the hybridoma supernatant binding to FSHR measured as fold-MFI K562-FSHR/K562. After the first round of screening, we went forward with the top 20 clones (left of the red bar)

Supp Figure 2

A

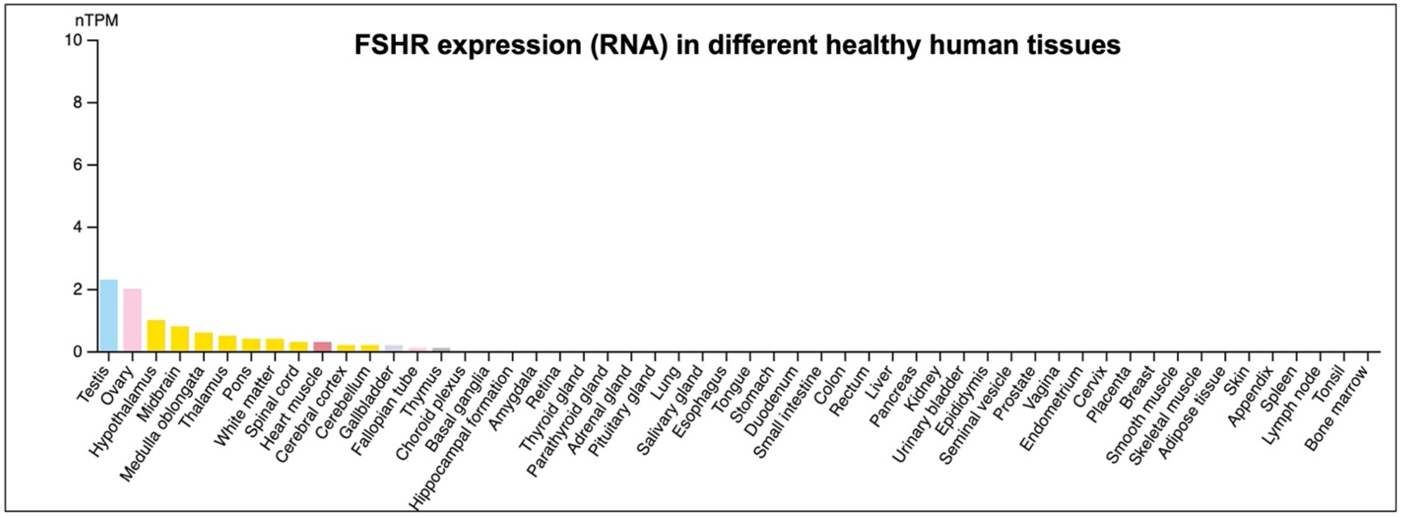

B

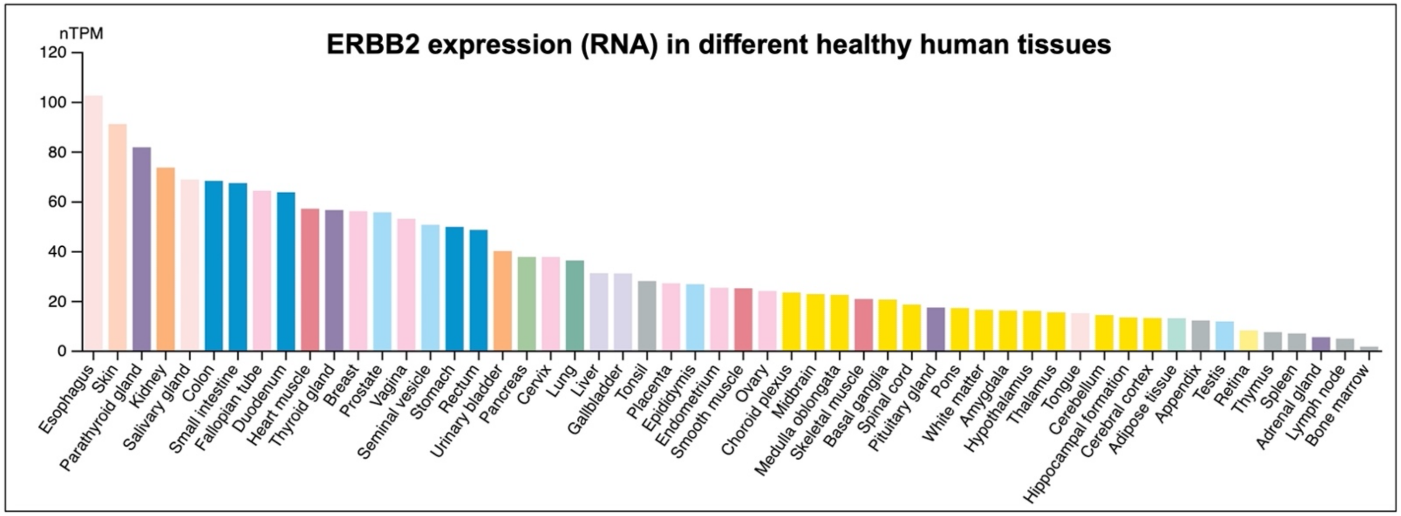

Supplementary Figure 2.

Differential expression of FSHR and ERBB2/Her2 in 55 tissue types. (A) Differential expression (RNA) of FSHR in 55 different human tissue types, as analyzed using 'The Human Protein Atlas' database. (B) Differential expression (RNA) of ERBB2/Her2 in different human tissues, as analyzed using 'The Human Protein Atlas' database (31); nTPM: normalized expression level.

Supp Figure 3

A

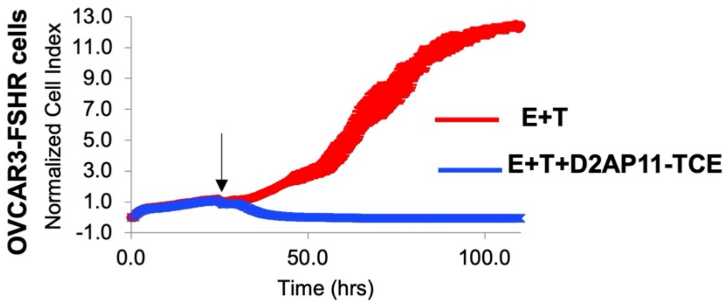

Effector cells: Human T cells
Target cells: OVCAR3-FSHR cells

B

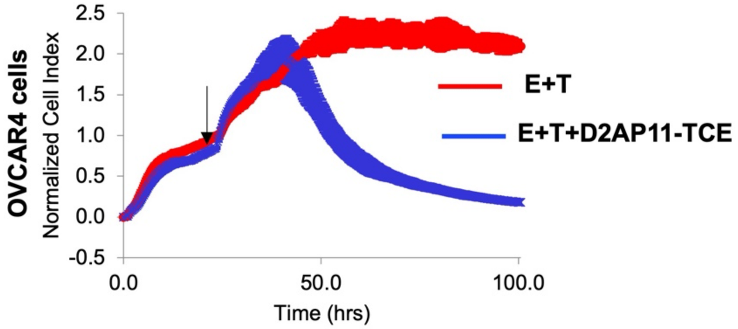

Effector cells: Human T cells
Target cells: OVCAR4 cells

C

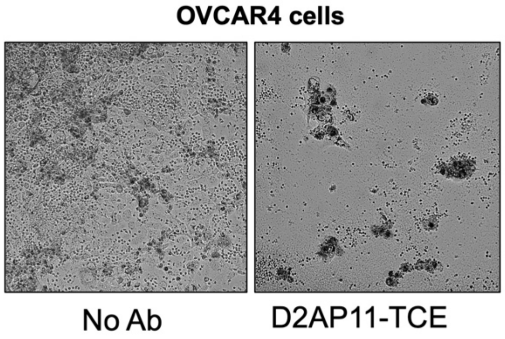

Supplementary Figure 3.

FSHR targeted bispecific T cell engagers induced ovarian cancer cytotoxicity in the presence of purified T cells. *In vitro* cytotoxicity resulting from co-culture of T cells with (**A**) OVCAR3-FSHR cells and (**B**) OVCAR4 cells in the presence of D2AP11-TCE. The real time *in vitro* cytotoxicity analysis was done by xCELLigence; E: T= 5:1/10:1 (**C**) Images showing the cytotoxic effect of D2AP11-TCE on OVCAR4 cells in the presence of human T cells, 3 days after the addition of effector cells and antibodies. No Ab (Only Effector+Target) served as control. Arrow indicates the time at which the effector cells and TCE were added.

Supp Figure 4

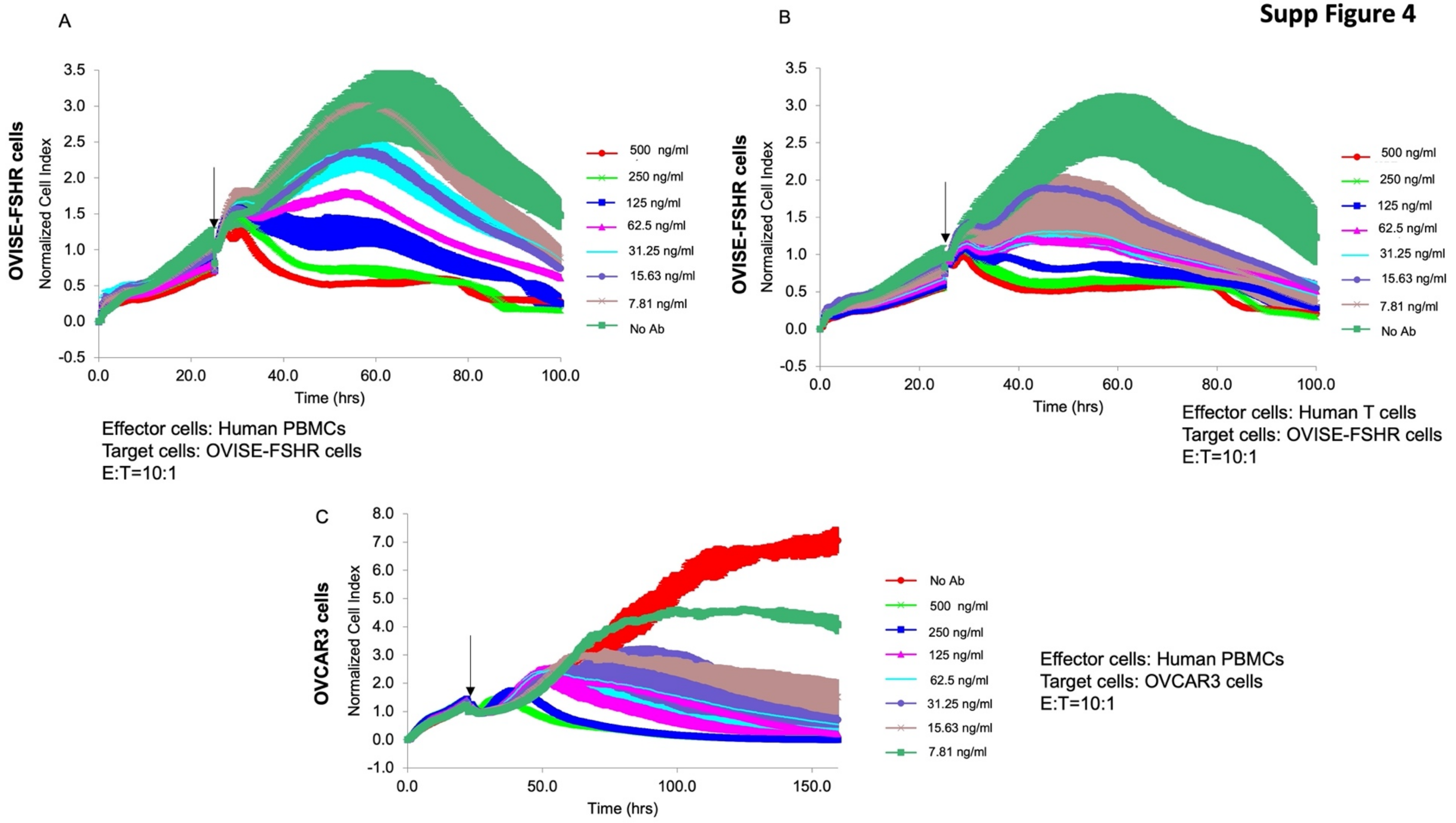

Supplementary Figure 4.

Dose dependent killing of OVISE-FSHR cells by D2AP11-TCE. Dose dependent killing observed in OVISE-FSHR cells by D2AP11-TCE in the presence of (**A**)Human PBMCs and (**B**)Human T cells. (**C**)Dose dependent killing of OVCAR3 cells by D2AP11-TCE in

the presence of Human PBMCs. The real time *in vitro* cytotoxicity analysis was done by xCELLigence; E: T= 10:1. D2AP11-TCE was added at the indicated concentrations ranging from 500 ng/ml to 7.81 ng/ml.

Supp Figure 5

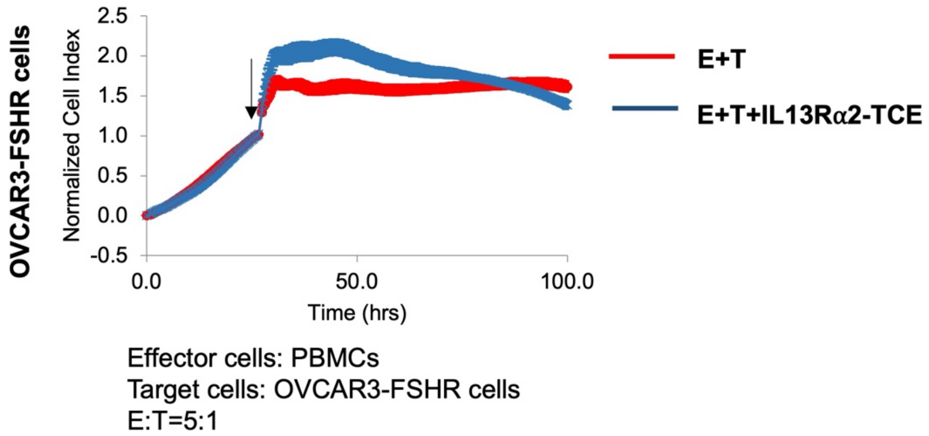

Supplementary Figure 5.

Irrelevant TCE did induce killing in FSHR overexpressing ovarian cancer cells. IL13Rα2-TCE (an irrelevant/non FSHR targeting TCE) did not cause cytotoxicity to FSHR overexpressing OVCAR3 cells in the presence of human PBMCs. Arrow indicates the time at which the effector cells and TCE were added. Red line indicates no Ab control (Only Effector +Target cells); E: T=5:1.

Supp Figure 6

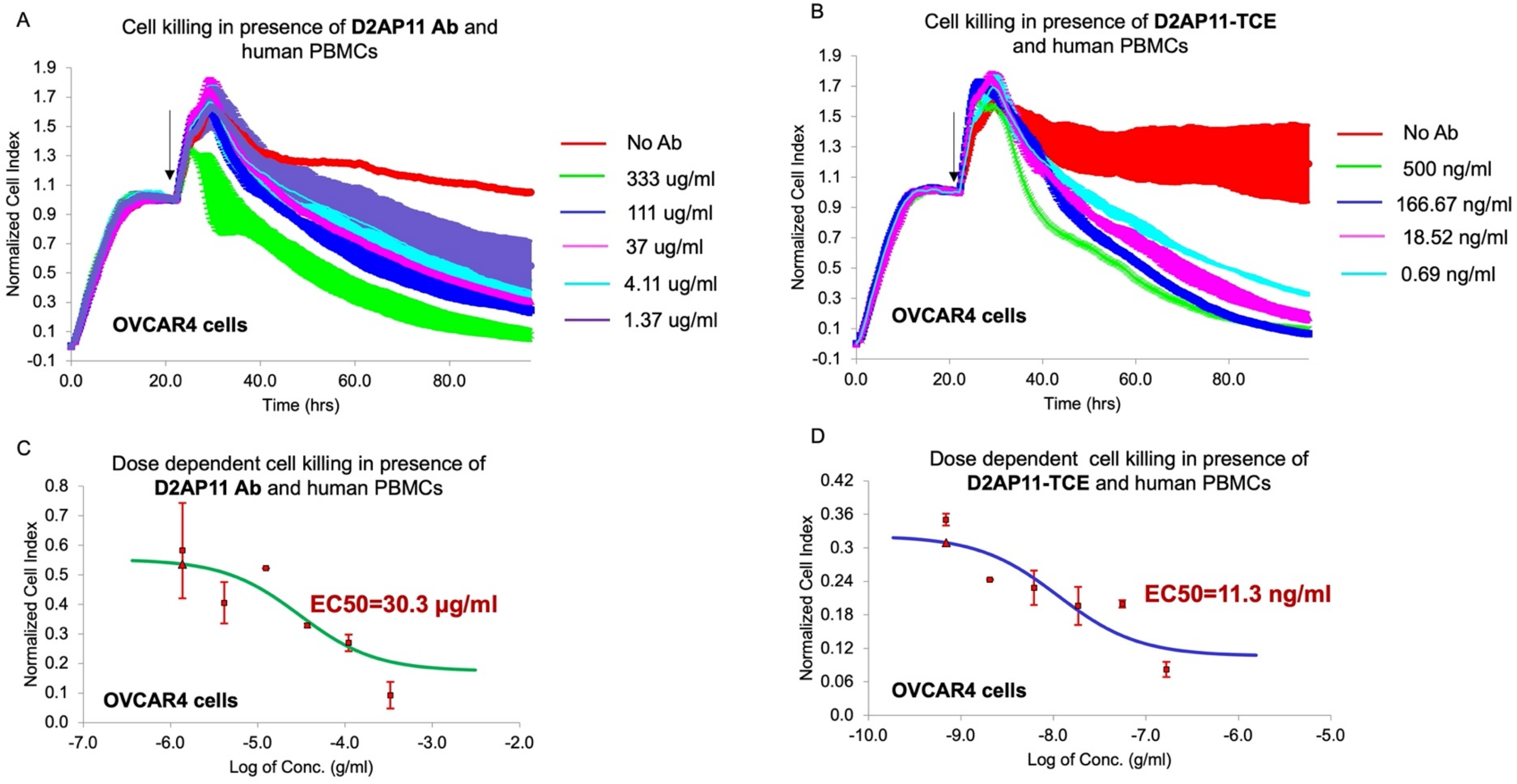

Supplementary Figure 6.

Comparative cell killing analysis of D2AP11 and D2AP11-TCE. (**A**)Killing of OVCAR4 cells by D2AP11 anti-FSHR antibody at the indicated concentrations in presence of human PBMCs. (**B**) Killing of OVCAR4 cells by D2AP11-TCE at the indicated

concentrations in presence of human PBMCs. Arrow indicates the time at which the effector cells and D2AP11/D2AP11-TCE was added. (**C**) D2AP11 anti-FSHR ab exhibited dose dependent killing of OVCAR4 cells with EC50 value of 30.3 μg/ml, in presence of human PBMCs (**D**) D2AP11-TCE displayed dose dependent killing of OVCAR4 cells with EC50 value of 11.3 ng/ml, in the presence of human PBMCs, indicating ~1000-fold higher potency compared to the anti-FSHR antibody. The real time *in vitro* cytotoxicity analysis was done by xCELLigence; E: T= 10:1. EC50 values were calculated using RTCA Pro software.
